# Risk Factors for Bunyavirus-Associated Severe Fever with Thrombocytopenia Syndrome: A Community-Based Case-Control Study

**DOI:** 10.1371/journal.pone.0166611

**Published:** 2016-11-15

**Authors:** Jian-li Hu, Zhi-feng Li, Xiao-chen Wang, Lei Hong, Hao He, Wei-guo Chen, Lu-xun Li, Ai-hua Shen, Xue-jian Liu, Shou-guo Yuan, Jian-gang Zhou, Wen-wen Tan, Wei-zhong Zhou, Fen-yang Tang, Feng-cai Zhu, Chang-jun Bao

**Affiliations:** 1 Department of Acute Infectious Disease Control and Prevention, Jiangsu Provincial Center for Disease Control and Prevention, Nanjing, Jiangsu Province, China; 2 Medical School, Nanjing University, Nanjing, Jiangsu Province, China; 3 Department of Acute Infectious Disease Control and Prevention, Nanjing Municipal Center for Disease Control and Prevention, Nanjing, Jiangsu Province, China; 4 Department of Disease Control and Prevention, Gulou District Center for Disease Control and Prevention, Nanjing, Jiangsu Province, China; 5 Department of Acute Infectious Disease Control and Prevention, Chuzhou Municipal Center for Disease Control and Prevention, Chuzhou, Anhui Province, China; 6 Department of Acute Infectious Disease Control and Prevention, Lishui District Center for Disease Control and Prevention, Nanjing, Jiangsu Province, China; 7 Department of Acute Infectious Disease Control and Prevention, Xuyi County Center for Disease Control and Prevention, Huai’an, Jiangsu Province, China; 8 Department of Acute Infectious Disease Control and Prevention, Yixing County Center for Disease Control and Prevention, Wuxi, Jiangsu Province, China; University of Texas Medical Branch, UNITED STATES

## Abstract

**Background:**

Severe fever with thrombocytopenia syndrome (SFTS) is an emerging infectious disease caused by a novel bunyavirus. Previous studies about risk factors for SFTSV infection have yielded inconsistent results, and behavior factors have not been fully clarified.

**Methods:**

A community-based, 1:4 matched case-control study was carried out to investigate the risk factors for SFTS in China. Cases of SFTS were defined as laboratory-confirmed cases that tested positive for real-time PCR (RT-PCR) for severe fever with thrombocytopenia syndrome bunyavirus (SFTSV) or positive for IgM antibodies against SFTSV. Controls of four neighborhood subjects were selected by matching for sex, age, and occupation. Standardized questionnaires were used to collect detailed information about their demographics and risk factors for SFTSV infection.

**Results:**

A total of 334 subjects participated in the study including 69 cases and 265 controls. The median age of the cases was 59.5 years, 55.1% were male, and 87.0% were farmers. No differences in demographics were observed between cases and controls. In the final multivariate analysis, tick bites two weeks prior to disease onset (OR = 8.04, 95%CI 3.34–19.37) and the presence of weeds and shrubs around the house (OR = 3.46, 95%CI 0.96–12.46) were found to be risk factors for SFTSV infection; taking preventative measures during outdoor activities (OR = 0.12, 95%CI 0.01–1.01) provided greater protection from SFTSV infection.

**Conclusions:**

Our results further confirm that SFTSV is transmitted by tick bites and prove that preventative measures that reduce exposure to ticks can prevent SFTSV infection. More efforts should be directed toward health education and behavior change for high-risk populations, especially outdoor workers, in SFTS endemic areas.

## Introduction

Severe fever with thrombocytopenia syndrome (SFTS) is an emerging infectious disease caused by a new member of the *Phlebovirus* species in the family *Bunyaviridae*., and was first discovered among the rural areas in the central and eastern regions of China in 2009 [1]. The major clinical manifestations of SFTS include high fever, gastrointestinal symptoms, thrombocytopenia, leukocytopenia. The average case-fatality rate was about 12% but could be as high as 30% for some populations [2, 3]. According to surveillance data released by China CDC, the geographic locations of SFTS cases have expanded to at least 23 provinces/municipalities in mainland China. Accordingly, the reported number of cases has increased remarkably from 461 in 2011 to 2,073 in 2015 [4]. SFTS cases were also reported in Japan, South Korea and North Korea [5–7]. Heartland virus and Hunter Island Group virus, which are both novel tick-borne phleboviruses and genetically related to, but distinctly different from the severe fever with thrombocytopenia syndrome bunyavirus (SFTSV), have been isolated from leukocytes of patients in the United States [8] and ticks in Australia [9], respectively. SFTSV and similar viruses pose an increasingly important challenge to global health.

Previous studies have demonstrated that the disease can be transmitted via direct contact with the blood or mucous of an SFTS patient [10, 11]. However, the primary means of transmission is generally believed to be by tick bites. Therefore, the health behaviors of individuals during outdoor activities, and the maintenance of clean dwelling environments are critical factors in controlling SFTS. Currently, little attention has been given to risk factors for the disease. Moreover, one hospital-based case control study and one sero-epidemiological survey on risk factors of SFTSV infection have yielded inconsistent results [12, 13]. Thus, we conducted a community—based case-control study to identify the risk factors of bunyavirus-associated SFTS and to complement the previous studies.

## Materials and Methods

### Ethics Statement

The protocol was approved by the Ethics Committee of the Jiangsu Provincial Center for Disease Control and Prevention (JSCDC), the governmental agency in charge of communicable disease control in Jiangsu Province. All aspects of the study complied with the Declaration of Helsinki. Written informed consent was obtained from all subjects involved in this study, and the dataset was analyzed anonymously.

### Case and Control Definition

The national guideline for prevention and control of SFTS was used as a reference for the case inclusion criteria [12]. Suspected SFTS patients were defined as persons who present with fever (temperature is ≥38°C) accompanied with thrombocytopenia and leukopenia. Confirmed SFTS patients were defined as suspected patients who tested positive for RT-PCR or positive for IgM antibodies against SFTSV. In this study, the case subjects were defined as confirmed SFTS cases from 2011 to 2013 in Jiangsu Province. Eligible control subjects were defined as neighbors of the case subjects who were matched by age (within three years), sex, and occupation (farmer or non-farmer), local residence time (longer than 6 months), and were negative for IgG antibodies against SFTSV to exclude unapparent and/or past SFTSV infection.

### Microbiological Analyses

Serum samples from suspected SFTS patients and identified matched controls were collected and transported in a cold box (4–8°C) to JSCDC’s laboratories for testing. RT-PCR detection was used to detect SFTSV RNA as previously described [14]. SFTSV-specific IgM antibodies in sera of suspected patients were examined with a commercial ELISA kit (Xinlianxin, Wuxi, China) according to the manufacturer’s instructions. SFTSV-specific IgG antibodies were detected in sera of controls by IFA as previously described [15].

### Data and samples collection

Trained interviewers visited the cases and identified matched controls (1:4 pair matching) from the cases’ nearest neighbors. Standardized questionnaires were used to collect detailed information on cases and controls. The study subjects were asked about their demographics (age, gender, home address, occupation), living and working environment (e.g. presence of rats, weeds and shrubs, and ticks), exposure history within the two weeks prior to fever onset (e.g. type of occupation, raising/contacting with animals, tick or other insect bites, and taking preventative measures during outdoor activities) and other possible risk factors (resting on a grass field, working with broken skin, and underlying diseases). The questionnaires of cases were completed within two weeks after laboratory diagnosis. All questionnaires were systematically verified by JSCDC study coordinators for data completeness.

### Statistical Analysis

Data was double entered into Epidata 3.02 (the EpiData Association, Denmark, Europe), and a database consistency check was performed. SPSS version 18.0 (Statistical Product and Service Solutions, Chicago, IL, USA) was used for all statistical analyses. Risk factors for SFTS were identified using univariate and multivariable conditional logistic regression models. Risk factors of univariate analysis with *P*≤0.10 were included in the multivariable model. The backwards stepwise elimination procedure was applied to exclude the variables with *P*>0.10 in the multivariable model. Consequently, we present odds ratios (ORs) with 95% confidence intervals (CIs) of exposure for various factors.

## Results

A total of 334 subjects participated in the study including 69 cases and 265 matched controls without refusals. 67 cases had positive results of PCR testing and 2 cases had only positive for IgM by ELISA testing.

All of the subjects’ ethnicity was Han Chinese. For cases and controls, the average age was 59.5 years and 60.4 years; 55.1% and 52.2% were male; 87.0% and 82.1% were farmers, respectively. There were no significant differences in demographic characteristics between cases and controls (Table 1).

**Table 1:** Demographic characteristics of the cases and controls.

characteristics	cases(n = 69)	controls(n = 265)	P-value
	n(%) or mean±SD	n(%) or mean±SD	
Age	59.5±11.5	60.2±11.5	0.618
Sex			
Male	38(55.1)	131(52.2)	0.698
Female	31(44.9)	120(47.8)	
Occupation			
Farmer	60(87.0)	206(82.1)	0.429
Non-Farmer	9(13.0)	45(17.9)	

SD: standard deviation.

In the univariate conditional logistic regression model(*P*≤0.10), the following factors were associated with a high risk of SFTSV infection: four environmental factors (presence of weeds and shrubs in working areas, presence of weeds and shrubs around the house, presence of ticks in working areas or around the house, and domestic animals illness or death two weeks prior to disease onset); and three behavioral risk factors (raising dogs, raising cattle, and tick bites two weeks prior to disease onset). Taking preventative measures during outdoor activities was associated with a reduced risk of being infected with SFTSV and was reported more often by controls (10.7%) than by cases (1.5%). Other factors were not significantly different between cases and controls (Table 2).

**Table 2:** Univariate conditional logistic regression analyses of potential risk factors.

Exposure factors	Cases	Controls	χ^2^	P-value	OR(95%CI)
	n(%)	n(%)			
Presence of weeds and shrubs in working areas					
Yes	64(92.8)	210(79.2)	3.42	0.072	2.66(0.92–7.71)
No	5(7.2)	55(20.8)			
Presence of weeds and shrubs around the house					
Yes	59(85.5)	198(74.7)	3.75	0.058	2.49(0.97–6.40)
No	10(14.5)	67(25.3)			
Presence of ticks in working areas or around the house					
Yes	49(71.0)	138(52.1)	4.91	0.030	2.35(1.09–5.09)
No	20(29.0)	127(47.9)			
Presence of rats in home					
Yes	63(91.3)	228(86.0)	0.96	0.330	1.62(0.61–4.30)
No	6(8.7)	37(14.0)			
Presence of rats in the working areas					
Yes	40(58.0)	140(52.8)	0.06	0.807	1.08(0.58–2.02)
No	29(42.0)	125(47.2)			
Farming					
Yes	59(85.5)	195(73.6)	2.04	0.157	1.78(0.80–3.97)
No	10(14.5)	70(26.4)			
Hunting					
Yes	3(4.3)	7(2.6)	0.01	0.918	1.08(0.24–4.85)
No	66(95.7)	258(97.4)			
Picking tea or herbs					
Yes	21(30.4)	57(21.5)	1.25	0.265	1.71(0.67–4.39)
No	48(69.6)	208(78.5)			
Raising dogs					
Yes	48(69.6)	135(50.9)	4.64	0.030	2.01 (1.06–3.82)
No	21(30.4)	130(49.1)			
Raising cats					
Yes	32(46.4)	107(40.4)	0.01	0.962	1.02(0.55–1.88)
No	37(53.6)	158(59.6)			
Raising cattle					
Yes	11(15.9)	21(7.9)	3.11	0.083	2.22(0.90–5.44)
No	58(84.1)	244(92.1)			
Raising goats					
Yes	4(5.8)	12(4.5)	0.02	0.886	1.09(0.33–3.56)
No	65(94.2)	253(95.5)			
Raising pigs					
Yes	15(21.7)	59(22.3)	1.54	0.217	0.60(0.26–1.35)
No	54(78.3)	206(77.7)			
Raising poultry					
Yes	57(82.6)	201(75.8)	0.11	0.736	1.14(0.53–2.49)
No	12(17.4)	64(24.2)			
Domestic animals illness or death					
Yes	6(8.7)	8(3.0)	3.25	0.083	2.77(0.88–8.74)
No	63(91.3)	257(97.0)			
Contacting with a dead rat					
Yes	6(8.7)	41(15.5)	0.91	0.344	0.61(0.22–1.69)
No	63(91.3)	224(84.5)			
Contacting with wild animals					
Yes	67(97.1)	236(89.1)	1.58	0.223	2.54(0.57–11.40)
No	2(2.9)	29(10.9)			
Removing ticks from domestic animals					
Yes	13(18.8)	37(14.0)	0.27	0.601	1.22(0.58–2.59)
No	56(81.2)	228(86.0)			
Tick bites					
Yes	30(43.5)	32(12.1)	35.86	<0.001	9.13(3.92–21.27)
No	39(56.5)	233(81.9)			
Other insects bites					
Yes	26(37.7)	93(36.5)	2.12	0.149	1.85(0.80–4.26)
No	43(62.3)	162(63.5)			
Taking preventative measures during outdoor activities^#^					
Yes	1(1.5)	25(10.7)	8.19	0.020	0.09(0.01–0.68)
No	65(98.5)	209(89.3)			
Resting on a grass field					
Yes	54(78.3)	176(66.4)	1.62	0.206	1.59(0.78–3.26)
No	15(21.7)	89(33.6)			
Working with broken skins					
Yes	12(17.4)	47(17.7)	0.36	0.547	1.30(0.55–3.07)
No	57(82.6)	218(82.3)			
Having underlying diseases					
Yes	20(29.4)	100(37.7)	0.76	0.383	0.76(0.41–1.41)
No	48(70.6)	165(62.3)			

^#:^ preventative measures included wearing gloves or boots, fastening cuffs and trouser mouths, wearing a long-sleeved shirt and using insect repellents.

All exposure factors were within the previous two weeks prior to disease onset. OR: odds ratio; CI: confidence interval.

In the multivariable conditional logistic regression model, two variables represented significant risk factors for SFTSV infection: tick bites two weeks prior to disease onset (OR = 8.04, 95%CI 3.34–19.37) and the presence of weeds and shrubs around the house (OR = 3.46, 95%CI 0.96–12.46); one variable of taking preventative measures during outdoor activities (OR = 0.12, 95%CI 0.01–1.01) was a significant protective factor for SFTSV infection (Table 3).

**Table 3:** Multivariate conditional logistic regression analyses of potential risk factors.

Exposure factors	Partial regression coefficient	Partial regression coefficient standard error	P-value	Adjusted OR	Adjusted OR 95.0% CI
Tick bites	2.08	0.45	<0.001	8.04	3.34–19.37
Taking preventative measures during outdoor activities	-2.16	1.11	0.051	0.12	0.01–1.01
Presence of weeds and shrubs around the house	1.24	0.65	0.058	3.46	0.96–12.46

All exposure factors were within the previous two weeks prior to disease onset. OR: odds ratio; CI: confidence interval.

## Discussion

Using a community-based case-control study, we identified three factors important to SFTSV infection. These include two risk factors: tick bites two weeks prior to disease onset, and the presence of weeds and shrubs around the house; and one protective factor, which is taking preventative measures during outdoor activities.

To our knowledge, the only comparable study that used hospitalized patients as controls found associations between SFTSV infection and risk factors such as tick bites two weeks prior to disease onset, owned cattle, owned cats, presence of ticks in living area, worked in the field, presence of weeds and shrubs in working areas. However, selection biases might have occurred due to the fact that farmers were more common among cases than controls in that study [12]. Another sero-epidemiological survey also tried to explore risk factors by analyzing determinants of SFTSV sero-prevalence among healthy participants, in which persons with positive SFTSV-specific IgG antibodies were cases and those with negative SFTSV-specific IgG antibodies were controls. They reported that raising goats, farming, and grazing might be risk factors for a healthy population, but excluded tick bites as a risk factor [13]. The results of this sero-epidemiological survey were controversial, because tick bites are known as one of the main forms of transmission. However, the nature of their study design may have resulted in selection and recall bias.

Compared with the above-mentioned previous studies, our study has specific strengths. First, the potential for selection bias was avoided, because the age, sex, occupation and neighborhood-matched design may have made cases and control subjects similar for certain variables, including residential area and possibly work place. Second, the potential for recall bias was small because of the use of new cases diagnosed within the past two weeks. Third, the study targeted behavior risk factors and the results have definite implications for public health because the controls were recruited from all disease-endemic areas and resided in the same surrounding areas as the cases. Fourth, the study was representative of the Chinese population because of the use of community-based study subjects.

In our study, tick bites two weeks prior to disease onset was the most significant risk factor, which is consistent with previous studies. Ticks, particularly *Haemaphysalis longicornis*, were thought to be the primary vector for SFTSV based on several lines of evidence as follows. First, a high proportion of patients diagnosed with SFTS reported a history of tick bites [1, 2, 16–18]. Second, spatial and temporal distributions of human cases were consistent with the fluctuation of certain species of ticks in a given endemic area [1, 2, 16, 19, 20]. Third, several research groups have detected SFTSV-specific nucleotide sequences, or isolated viruses from ticks collected from animals, or the environment, which have high homology (93–100%) with SFTSV isolated from patients [1, 2, 19, 21–24]. Finally, ticks can acquire the virus by feeding on blood of the SFTSV-infected host animals and transstadially and transovarially transmit it to other developmental stages of ticks. SFTSV infected ticks can transmit the virus to host animals during feeding [24, 25]. The other identified risk factor of weeds and shrubs present around the house is consistent with the fact that *Haemaphysalis longicornis* is known as a bush or scrub tick that is free-living in the environment waiting a suitable host (e.g. small mammals, domestic animals and wildlife). This further supports the belief that ticks are the main vectors of transmission. Furthermore, according to our research data, the fact that 76.9% of overall subjects have weeds and shrubs around their houses demonstrates that the sanitary condition of the environment in disease-endemic areas is very poor.

Our results show that taking preventative measures during outdoor activities while wearing gloves or boots, fastening cuffs and trousers mouths, wearing a long-sleeved shirt, and using insect repellents were protective factors with statistical significance, suggesting that those who tend to expose their skin during outdoor activities are more vulnerable to SFTSV infection. Only 8.7% of overall subjects in our study had taken preventative measures listed above. To date, there is no vaccine against SFTSV infection available. Therefore, strengthening and promoting health education and behavior change in endemic areas is critical for high-risk populations, especially outdoor workers. The risk of tick to human transmission can be minimized by, the use of gloves and minimal exposure of bare skin to tick-infested vegetation and animals; the use of commercially available insect repellents, including diethyltoluamide on bare skin; removing weeds and shrubs around the house to eliminate tick habitats; and treating the livestock with acaricides to decrease the population of infected ticks [26].

Person-to-person transmission of SFTSV may be possible because the infection has been reported in some family clusters [10, 11]. However, all of the cases in the study were sporadic and not linked epidemiologically, so we did not analyze the variable of “contact with blood or mucous of an SFTS patient”. One study found that there was no statistically significant difference for seroprevalence of SFTSV antibodies between those who had been in close contact with an SFTS patient and those who had not [27], suggesting that person-to-person transmission is not the main risk factor for infection, and this mode of transmission is probably limited.

Our study also had limitations. First, the sample size was relatively small considering the heterogeneity of the study participants. Second, we could not identify a dose-response relationship between the frequency of tick bites and the occurrence rate of infection. Consequently, the efficacy of detailed preventative measures was not clearly evaluated because the sample size of the participants with the same specific measures was very small.

In conclusion, our study further confirmed that SFTSV is transmitted by tick bites and proved that preventative measures that reduce exposure to ticks can prevent SFTSV infection. We should pay greater attention to promoting health education and behavior change among high-risk populations, especially outdoor workers, in the SFTS endemic area.

## Supporting Information

S1 Table Detailed demographics and risk factors for SFTSV infection of the cases and controls in the JS2011-2013 Database. (XLSX)
